# Fear of Missing out and Online Social Anxiety in University Students: Mediation by Irrational Procrastination and Media Multitasking

**DOI:** 10.3390/bs15010084

**Published:** 2025-01-18

**Authors:** Weimiao Wu, Jie Zhang, Namjeong Jo

**Affiliations:** 1 School of Education, Woosuk University, Jeonju 55338, Republic of Korea; alisamiao@stu.woosuk.ac.kr; 2 Department of Psychology, Hebei Normal University, Shijiazhuang 050024, China

**Keywords:** FoMO, online social anxiety, irrational procrastination, media multitasking, university students

## Abstract

With the rapid growth of internet mobile technology, recent research has increasingly focused on the mental health challenges faced by young people, particularly in relation to social media use. One significant concern is the impact of the fear of missing out (FoMO) and online social anxiety, yet the underlying mechanisms that link these factors remain largely unexplored. This study aims to address this gap by investigating the role of FoMO in predicting online social anxiety among university students, with a particular focus on understanding how irrational procrastination and media multitasking may mediate this relationship. In total, 451 university students completed a survey on demographics, FoMO, online social anxiety, irrational procrastination, and media multitasking questionnaires. After controlling for demographic variables, the findings revealed that (a) FoMO showed a significant positive correlation with online social anxiety; (b) the connection between FoMO and online social anxiety in university students was partially mediated by irrational procrastination; and (c) the connection between FoMO and online social anxiety in university students was partially mediated by media multitasking. This research contributes to the understanding of the psychological mechanisms that link FoMO to online social anxiety, offering insights that can inform interventions aimed at improving university students’ mental health in the digital age.

## 1. Introduction

With the rapid growth of mobile technology, more than half of the global population now has access to the Internet via smartphone (Statista, 2024). As the time and frequency with which people access social media via smartphones increases, they are very convenient for our life interactions, while also having some negative consequences, causing psychological disturbances, irritability, or panic, such as fear of missing out (FoMO) (Song et al., 2017). FoMO has attracted significant scholarly interest as a new phenomenon emerging from the modern social media world (Fioravanti et al., 2021). Indeed, young people experience higher degrees of FoMO on social media (Casale & Fioravanti, 2020; Elhai et al., 2018; Przybylski et al., 2013). In China, a survey of young people revealed that the proportion is 78.3%, with severe FoMO reaching 15.2% (Chai et al., 2018). As networked and intelligent processes evolve, FoMO will become increasingly widespread (Akbari et al., 2021).

Previous studies have found that young people who experience high levels of FoMO may develop a tendency for excessive social media usage, potentially leading to addictive behaviors (Elhai et al., 2016). This tendency has a negative influence on university students’ mental health, contributing to increased risks of depression, anxiety, and obsessive–compulsive symptoms (Andreassen et al., 2016; Elhai et al., 2020), as well as lower levels of psychological well-being (e.g., life satisfaction) and self-esteem (Alt, 2018; Błachnio & Przepiorka, 2019). Moreover, individuals experiencing higher levels of FoMO tend to exhibit lower interpersonal security and engage in fewer prosocial behaviors (Luo et al., 2024). More importantly, one study found that FoMO predicts an individual’s online social anxiety (Jiang et al., 2020).

Online interactions have become an effective alternative to face-to-face interactions for socially anxious groups due to its advantages of flexibility and anonymity (Shepherd & Edelmann, 2005; Young & Lo, 2012). With the gradual blurring of the boundaries between online and offline interactions, social media situations can also induce social anxiety and even cause it to develop into new forms of anxiety (Davidson & Farquhar, 2014). In the United States alone, 17% of university students report feeling social anxiety from using social media (Campisi et al., 2012). Online social anxiety, as a negative emotional experience, can hinder normal social interactions in university students and affect their overall mental health (Chen et al., 2020b). As a result, there is a need to investigate in depth how FoMO influences university students’ online social anxiety, as well as the potential mediating variables involved in this relationship. Understanding these mechanisms is crucial for developing effective interventions to improve university students’ mental health in the digital age.

## 2. Literature Review

### 2.1. FoMO and Online Social Anxiety

FoMO is a psychological trait defined as “a pervasive apprehension that others might be having rewarding experiences from which one is absent” (Przybylski et al., 2013, p. 1841). It is a state of being “left behind” (Salem, 2015), fear of falling behind in current affairs, fear of not being the first to catch up with what peers are up to to the point of frequently refreshing social media platforms, and so on (Chai et al., 2018). According to the compensatory internet use theory, stressful life events may increase the probability of participating in activities such as internet surfing to alleviate negative emotions (Akbari et al., 2021; Kardefelt-Winther, 2014). However, excessive internet use does not truly relieve stress (Beyens et al., 2016). Previous studies have revealed that FoMO among students correlates with more frequent internet use (Alt & Boniel-Nissim, 2018). Although social media use may have positive consequences (e.g., social support), excessive use driven by FoMO can, in turn, trigger more negative emotions (e.g., online social anxiety) in university students (Baker et al., 2016; Hunt et al., 2018; Jiang et al., 2020).

Online social anxiety is defined as negative interpersonal experiences, including tension, anxiety and fear, that individuals have during social media interactions (Chen et al., 2020b). Although online social anxiety has its unique characteristics (e.g., privacy concerns), it is still a branch of social anxiety and an extension and developed version of real-life social anxiety in the digital world (Chen et al., 2020a). Therefore, there is a significant transduction impact between real-life social anxiety and online social anxiety (Liu et al., 2022). For university students with high levels of social anxiety, reliance on online self-disclosure as a means of building social relationships may increase susceptibility to online social anxiety (Liu et al., 2022). This appears because individuals with high social anxiety are prone to social isolation in real life, and they may seek to fulfill their interpersonal needs online, perceiving online interactions as more secure than face-to-face communication (Lee & Stapinski, 2012).

FoMO is regarded as a stable personality trait (Buglass et al., 2017; Przybylski et al., 2013). A recent study found that specific personality traits, such as the fear of missing out on information, may increase emotions of uncertainty and anxiety within virtual social environments (Zhu et al., 2024). Such feelings cause pathological compensation for online social behaviors, more frequent updating and checking of online social information, and an over-expenditure of one’s physical and mental resources on online social activities (Wang et al., 2021), all of which can contribute to increased levels of online social anxiety. The relationship between FOMO and online social anxiety and the mediating mechanisms associated with it have been discussed in existing studies (Duan et al., 2020). This paper will discuss the mediating role of other variables, such as procrastination, between them. According to the preceding discussion, we propose the first hypothesis in this study:

**Hypothesis 1.**  *FoMO can positively predict online social anxiety in university students.*

### 2.2. Irrational Procrastination as a Mediator

Steel (2007) describes procrastination as the irrational postponement of a task, as well as the voluntary postponement of an originally planned task, even though the delay may exacerbate the need to fulfil expectations. Solomon and Rothblum’s (1984) study mentioned that most college students have problems with procrastination and that the tendency to procrastinate becomes worse as they spend more time in college. According to neurobiological viewpoints, long-term intentions are replaced by impulses generated by the limbic system, which is especially susceptible to specific stimuli that provide immediate gratification (Kahneman, 2003; McClure et al., 2007). According to self-determination theory, FoMO can be viewed as a deficit in self-regulation due to unfulfilled psychological demands (Przybylski et al., 2013). Individuals with high levels of FoMO can easily obtain immediate pleasure from social media, which attracts their attention and distracts them from their initial plans (Steel, 2010). This subsequently leads to procrastination. A cross-sectional study found that FoMO strongly predicts irrational procrastination, which supports this hypothesis (Li & Ye, 2022).

Numerous studies have confirmed that procrastination is associated with various adverse psychological outcomes. For example, procrastination leads to lower self-esteem (Solomon & Rothblum, 1984); it also has a significant positive correlation with perceived stress, negative life events, and daily difficulties (Ferrari et al., 1995). Notably, social anxiety has been identified as a factor leading to procrastination in the existing literature (Petwal et al., 2021). However, this paper explores the possibility that procrastination may also exacerbate social anxiety. Some studies suggest that procrastination is a form of initial, temporary relief for unpleasant emotions, delaying the task and thus achieving it (Pychyl & Sirois, 2016). However, over time, it can instead exacerbate negative emotions (Sirois & Pychyl, 2013). Thus, social anxiety can be both a cause and a consequence of procrastination. Individuals who tend to procrastinate are more likely to develop self-doubt and excessive concern about others’ evaluations in social scenarios, exacerbating social anxiety. Given the strong transduction from social anxiety to online social anxiety, it is reasonable to suspect that procrastination may contribute to online social anxiety. Therefore, the following hypothesis is proposed:

**Hypothesis 2.**  *FoMO may indirectly influence online social anxiety in university students through the mediating role of irrational procrastination.*

### 2.3. Media Multitasking as a Mediator

The growth of media has facilitated the simultaneous completion of multiple media tasks. Media multitasking refers to simultaneous engagement in multiple electronic device-based media activities, such as completing academic tasks and using social media simultaneously or listening to music while browsing social media (Foehr, 2006). It has become the most common media usage behavior among young people (Carrier et al., 2009; Jeong & Fishbein, 2007). Research has shown that people engage in additional media tasks to regulate their mood and obtain emotional satisfaction. For example, multitasking positively correlates with enjoyment (Srivastava, 2013); boredom-induced fatigue and anxiety can also trigger multitasking behavior (Wang et al., 2006). Shin & Kemps (2020) hypothesized that media multitasking could be a behavioral strategy for avoiding negative emotional stimuli. Thus, in this context, FoMO serves as a negative emotional stimulus and is suggested as a potential predictor of media multitasking behavior (Popławska et al., 2021).

Different types of multitasking can affect social anxiety in various ways. For example, students seem to learn more efficiently when they switch between different academic tasks. In fact, experimental results from one study showed that participants spent more time completing tasks performed simultaneously than performing them sequentially (Rubinstein et al., 2001). Frequent media multitasking was distracting, and attentional control, in turn, was significantly associated with levels of anxiety and depression (Li & Fan, 2022). Furthermore, when students engage in media multitasking to relieve academic stress and anxiety (e.g., listening to music and swiping through social media), while it may temporarily alleviate negative emotions, in the long run, it interferes with problem-solving and further increases an individual’s depression and anxiety (Shin & Kemps, 2020). Thus, frequent media multitasking may be associated with social anxiety. Becker et al. (2013) proposed that increasing media multitasking has evolved into a distinct predictor of social anxiety and depression. This study supports this hypothesis.

**Hypothesis 3.**  *FoMO may indirectly influence online social anxiety in university students through the mediating role of media multitasking.*

### 2.4. The Present Study

Few studies have examined the specific internal mechanisms that link FoMO to online social anxiety. This study addresses a significant research gap in the previous literature by investigating the mediating role of irrational procrastination and media multitasking. A recent study investigated the mediating and moderating effects of positive and honest self-presentations on FoMO and online social anxiety (Duan et al., 2020). This research seeks to explore this mediating mechanism from a different perspective. Both irrational procrastination and media multitasking are seen as short-term protective mechanisms for coping with negative emotions. Still, excessive procrastination and frequent media multitasking behaviors are maladaptive coping styles that may ultimately exacerbate negative emotions (Shin & Kemps, 2020; Sirois & Pychyl, 2013).

**Figure 1:**
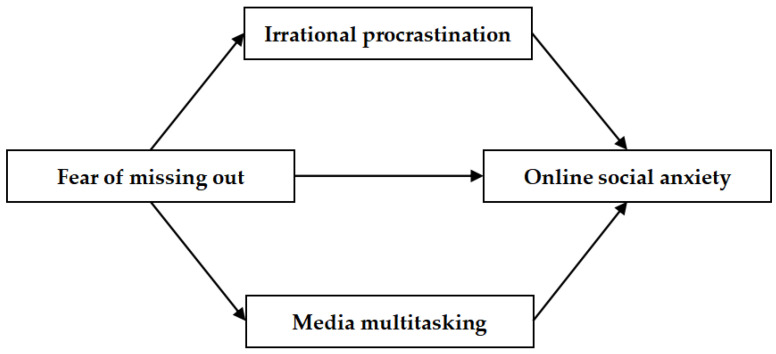
The proposed mediation model.

Therefore, this study proposes a mediation model that takes into account two maladaptive avoidance strategies of social media usage in university students: media multitasking and procrastination. The present study will address the following questions: RQ1, does FoMO positively predict online social anxiety? RQ2, does irrational procrastination serve as a mediator between FoMO and online social anxiety? RQ3, does media multitasking serve as a mediator between FoMO and online social anxiety (Figure 1)?

## 3. Method

### 3.1. Participants

This research used a convenient random sampling method to collect data. First, university students were randomly selected from Hebei Normal University, China. We then encouraged participants who engaged with this study to send the link to other university students. Each IP address was permitted single access to the survey to avoid data duplication. We also identified participants’ locations through their IP addresses. Ultimately, we collected samples from 27 provinces in China, primarily from Hebei, Guangdong, Jiangsu, and Zhejiang. Consent was obtained from all participants, who were invited to complete the questionnaires anonymously.

A total of 496 university students completed the survey. Upon excluding unqualified responses (e.g., some university students did not fill out the survey properly or failed to complete it), we ultimately gathered 451 valid responses for an effective response rate of 90.93%. Respondents’ average age was 20.50 years (ranging from 18 to 25, with SD = 1.32), and the group included 45 (10%) freshmen, 198 (43.90%) sophomores, 99 (22%) juniors, 109 (24.20%) seniors; among these, 283 were females (62.70%) and 168 were males (37.30%).

### 3.2. Research Tools

#### 3.2.1. Fear of Missing out Scale

The revised Chinese version of the Fear of Missing Out Scale (FoMOs) was utilized (Przybylski et al., 2013; Wang et al., 2018). This scale consists of a single dimension with 10 items. Participants were directed to complete the survey utilizing a 5-point Likert scale with options from “1 = not at all true of me” to “5 = extremely true of me”. An average score was computed across all ten items, with higher values reflecting a greater degree of FoMO. Internal consistency was assessed using Cronbach’s alpha, with a result of 0.86. 

#### 3.2.2. Irrational Procrastination Scale

The Chinese version of the Irrational Procrastination Scale (IPS) was applied (Ni et al., 2012; Steel, 2010). A total of 9 items were included, all categorized under a single category. The items were assessed utilizing a 5-point Likert-type scale from 1 (strongly disagree) to 5 (strongly agree). Three reverse-scored items were recorded. A higher total score indicates a greater degree of irrational procrastination. Internal consistency was assessed using Cronbach’s alpha, with a result of 0.77.

#### 3.2.3. Media Multitasking Index

The Chinese version of the Media Multitasking Index (MMI) was adopted (Ophir et al., 2009; Yang & Zhu, 2014). The questionnaire was divided into two sections. In Part 1, participants were asked to report separately the average time spent on 10 different types of media tasks per day: face-to-face talking, watching TV and movies, instant messaging or emailing, using social sites, using non-social text-oriented sites, talking on the telephone or video chatting, listening to music, print media, playing video or online games, and engaging in homework. In Part 2, participants indicated the extent to which they engaged in other media activities alongside their primary media task, which was assessed using a four-point Likert scale: never (0), occasionally (0.33), often (0.67), and always (1).

These data are used to calculate the media multitasking index (MMI) by using the formula *MMI* = $\sum_{\;i=1}^{\;i=10}\frac{m_{i\times h_{i}}}{h_{total}}$ (Ophir et al., 2009). In this formula, $h_{i}\;$is based on the reported number of hours per day using the primary media task i, $h_{total}$ is the total number of hours per day using the ten primary media tasks, and $m_{i}$ is an estimate of the amount of time participants reported engaging in the primary media while using other media activities. The media multitasking index was calculated to discriminate between groups of heavy and light media multitaskers (Ophir et al., 2009). Internal consistency was assessed using Cronbach’s alpha, with a result of 0.91.

#### 3.2.4. Online Social Anxiety Scale

This study utilized the revised version of the Social Anxiety Scale for Social Media Users (SAS-SMU) (Alkis et al., 2017; Chen et al., 2020a). The scale comprised 20 items organized into three sub-dimensions: 10 items on “fear of evaluation”, 6 items on “interaction anxiety”, and 4 items on “privacy concern”. A 5-point Likert scale assessed participants’ agreement with each item, with 1 indicating ‘strongly disagree’ and 5 indicating ‘strongly agree’. Higher scores reflected a greater level of online social anxiety. Internal consistency was assessed using Cronbach’s alpha, with a result of 0.94.

### 3.3. Statistical Analysis

The data analyses were conducted using IBM SPSS Statistics for Windows, version 26.0 (IBM Corp., Armonk, NY, USA). First, skewness and kurtosis needed to be analyzed to check the assumption of normality of the data and to check the data for multicollinearity issues through tolerance and VIF values. Second, descriptive statistics (mean and standard deviation) and Pearson correlations were conducted for the independent variable (FoMO), mediating variables (irrational procrastination, media multitasking), and the dependent variable (online social anxiety). Then, the mediating effects of irrational procrastination and media multitasking were assessed separately using Model 4 of the PROCESS macro for SPSS (Hayes, 2017). Finally, a bias-corrected bootstrap method with 5000 random samples was employed to evaluate the mediation hypotheses (Hayes, 2017). To ensure a more accurate relationship between the independent and dependent variables, the model controlled for gender and grade as covariates. Mediation was considered significant when the confidence interval (CI) around the indirect effect excluded zero.

## 4. Results

### 4.1. Common Method Deviation

Self-reported data collection methods may result in common methodological biases. A single-factor confirmatory factor analysis was conducted to evaluate common method bias before data processing (Podsakoff et al., 2003). The unrotated factor analysis results revealed the emergence of eleven factors with eigenvalues exceeding one, which together accounted for 59.60% of the total variance. The first main factor accounted for 27.38% of the variance, a value below 40% (Lee et al., 2011). This suggests that common method bias was not an issue.

### 4.2. Descriptive Statistics and Related Analysis for Each Variable

Skewness values for all variables ranged from −0.04 to 0.40, while kurtosis values ranged from −0.72 to −0.02. According to Kim (2013), these values were indicative of a normal distribution. Subsequent testing revealed that all predictor variables had tolerance values exceeding 0.30 and VIF values below 5. This indicates that there were no serious problems with multicollinearity (Kock, 2015).

Table 1 displays the mean and standard deviation for each variable, along with the Pearson correlations among all variables. The correlation matrix table indicates significant positive correlations among FoMO, irrational procrastination, media multitasking, and online social anxiety in university students (*p* < 0.01).

### 4.3. Testing for the Mediation Effect

The PROCESS macro for SPSS (Model 4) was used for mediation analysis (Hayes, 2017). A multiple mediation analysis examined the effect of FoMO on online social anxiety, with irrational procrastination and media multitasking as mediators while controlling for gender and grade. The models with irrational procrastination and media multitasking as mediators were significant, as shown in Table 2. The findings indicated that FoMO significantly predicted online social anxiety (*β* = 0.51, *p* < 0.001), as well as irrational procrastination and media multitasking (*β* = 0.35, *p* < 0.001; *β* = 0.10, *p* < 0.001). Additionally, irrational procrastination and media multitasking significantly predicted online social anxiety (*β* = 0.38, *p* < 0.001; *β* = 0.54, *p* < 0.01).

**Figure 2:**
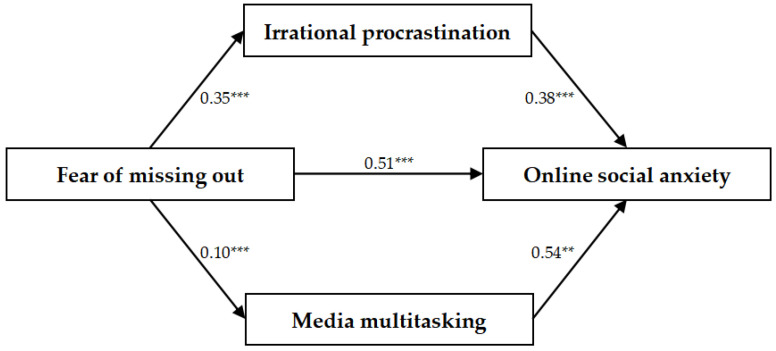
Standardized regression coefficients for the relationship between FoMO and online social anxiety as mediated by irrational procrastination and media multitasking. Note: ** *p* < 0.01 and *** *p* < 0.001.

Then, a bootstrap analysis employing the bias-corrected non-parametric percentage method was conducted to investigate the mediating effects further. Table 3 indicates that the direct effect of FoMO on online social anxiety was statistically significant (*p* < 0.001). Therefore, irrational procrastination and media multitasking partially mediated the interaction between FoMO and online social anxiety. Specifically, the mediating effect included two separate pathways: indirect pathway 1 (FoMO → irrational procrastination → online social anxiety) and indirect pathway 2 (FoMO → media multitasking → online social anxiety). The effect values for these pathways were 0.19 and 0.08, respectively. The 95% confidence intervals for both pathways excluded zero, confirming the mediation effect’s significance (see Figure 2 for the pathway model).

## 5. Discussion

This research examined the predictive influence of FoMO on university students’ online social anxiety, emphasizing the mediating effects of irrational procrastination and media multitasking. The results show that FoMO could positively predict online social anxiety in university students, and irrational procrastination and media multitasking mediates this relationship. The subsequent sections will provide a detailed examination of each hypothesis.

### 5.1. The Relationship Between FoMO and Online Socila Anxiety

The results show that university students’ FoMO significantly and positively predicted online social anxiety. The higher the degree of FoMO, the more severe the online social anxiety, which confirms Hypothesis 1. FoMO refers to a psychological phenomenon of a fear of missing out on social experiences or interactions (Przybylski et al., 2013), whereas online social anxiety is a negative emotional experience during social media interactions (Chen et al., 2020b). Research has shown a strong link between FoMO and online social anxiety (Jiang et al., 2020). Specifically, FoMO has been found to lead to excessive or addictive social media use (Alt, 2015; Elhai et al., 2016; Fuster et al., 2017). One of the primary mechanisms by which FoMO influences social anxiety is through social comparison theory, whereby individuals continually assess themselves in light of the perceived experiences of others (Wood, 1989). University students are in the stage of craving for recognition and affirmation from others, and university students with high levels of FoMO will frequently refresh social media for fear of missing out on the dynamics of their peers (Beyens et al., 2016). In this process, it is likely that comparisons with others lead to negative evaluations of the self, which in turn creates anxiety about their online social interactions and the information they share. University students with high levels of FoMO tend to experience multiple types of online social anxiety, such as content-sharing anxiety, interaction anxiety, privacy concerns, and self-assessment anxiety (Chen et al., 2020b). Thus, FoMO prompts individuals to be more sensitive to perceived social comparisons and self-expression concerns, which leads to greater vigilance about social media interactions and content sharing, ultimately exacerbating online social anxiety.

Schools and parents should guide university students to recognize the possible adverse effects of FoMO and reduce unnecessary anxiety. They should include social media use and mental health content in health education courses to help them reduce their dependence on social media. While guiding university students on personal career planning, they should also guide them to develop positive perceptions and self-confidence by realizing realistic and feasible goals and avoid undermining self-confidence by excessive comparisons with others.

### 5.2. The Mediating Role of Irrational Procrastination

As we hypothesized, we found that irrational procrastination mediated the link between FoMO and online social anxiety to some extent. Specifically, FoMO was positively associated with irrational procrastination during the first part of the mediation process. Consistent with previous research, university students experiencing high levels of FoMO tended to prioritize immediate gratification from digital interactions, leading to more cognitive failure behaviors and a reduced ability to focus on academic tasks, and, in turn, contributing to the tendency to procrastinate (Li & Ye, 2022). However, procrastination is not only a reactive response to information overload but can also be used as a maladaptive coping strategy to manage underlying emotional discomfort. Ferrari (1991) found that procrastination was associated with maladaptive tendencies; in another study, anxiety about others’ evaluations, perfectionism, and lack of self-confidence were reported to be the most significant causes of procrastination (Solomon & Rothblum, 1984). This provides new perspectives for exploring the causes of procrastination.

For the second part of the mediation process, irrational procrastination was positively associated with online social anxiety among university students. Previous studies have repeatedly shown that frequent procrastinators have poorer mental health than infrequent procrastinators (Ferrari, 1991; Stead et al., 2010). Notably, frequent procrastinators often engage in avoidance behaviors that not only affect their academic performance (Kim & Seo, 2015), but also impede their emotional regulation and coping skills, leading to lower self-esteem and higher anxiety (Hutchison et al., 2018; Solomon & Rothblum, 1984). This avoidance is exacerbated by procrastination behaviors in social media environments that impede the achievement of realistic tasks. As a result, university students may experience increased feelings of self-doubt and social anxiety. Furthermore, the link between procrastination and online social anxiety is not only correlated but likely driven by a more complex bidirectional relationship, as suggested by previous research and our findings (Petwal et al., 2021). Frequent procrastinators may avoid academic tasks or real-world social activities in favor of online interactions that give them a greater sense of control or escape, but this creates a cycle of social withdrawal and high anxiety in cyberspace. This cycle can be understood through self-determination theory (Deci & Ryan, 2004), where a lack of fulfillment of basic psychological needs such as competence, relevance, and autonomy may lead to procrastination and social anxiety.

Thus, our study highlights the importance of addressing both procrastination and FoMO in interventions aimed at reducing online social anxiety in university students, suggesting that addressing irrational procrastination may be a critical step in breaking the vicious cycle of anxiety exacerbated by social media use.

### 5.3. The Mediating Role of Media Multitasking

Consistent with our hypothesis, media multitasking is another explanatory mechanism for how FoMO is related to online social anxiety in university students, which validates Hypothesis 3. Specifically, for the first part of the mediation process, FoMO was positively related to media multitasking. This is consistent with the hypothesis from previous research that FoMO may be a predictive personality trait for media multitasking (Popławska et al., 2021). One interpretation is that media multitasking is a behavior that strategically responds to one’s emotions (e.g., FoMO). This is supported by the vigilance-avoidance hypothesis, which suggests that anxious individuals engage in “avoidance” behaviors in response to negative stimuli after initially focusing on them (Mogg et al., 2004). Emotion-oriented coping focuses attention on the source of the stress. In contrast, avoidance-oriented coping (e.g., media multitasking) diverts attention away from the source of stress (Shin & Kemps, 2020). However, a coping strategy such as media multitasking, although effective in the short term, is considered maladaptive in the long term. It decreases university students’ efficiency in dealing with problems and their ability to resolve underlying emotional issues, thus increasing psychological stress (Shin & Kemps, 2020). Therefore, in the second part of the mediation process, we concluded that media multitasking positively correlates with online social anxiety among university students. In support of this, Becker et al. (2013) found in a large-scale survey of 3019 undergraduates that frequent media multitasking behaviors significantly predicted social anxiety, even when controlling for other factors such as general media use and personality traits. This finding suggests that frequent media multitasking is a unique behavioral mechanism that leads to increased anxiety (Becker et al., 2013). That is, frequent engagement in multitasking may lead to cognitive overload, making individuals more susceptible to anxiety, including specific forms of online social anxiety such as content-sharing anxiety and self-evaluation anxiety. This may create a vicious cycle in which individuals seeking relief from FoMO-type anxiety engage in more media multitasking, only to experience intense social disconnection, depression, and anxiety both online and offline.

As adolescents enter university life, a critical period of physical and mental development, school mental health education should focus on guiding college students to establish correct media use behaviors and recognize the dangers of excessive media multitasking. Regular social skills training should be conducted to help university students improve their emotion management and interpersonal skills, reduce their over-reliance on virtual socialization, and avoid negative emotions exacerbated by excessive media multitasking.

### 5.4. Limitations

This study has the following limitations. First, this study’s cross-sectional research method limits the causality inference from the results. A longitudinal design should be used in future to examine the causal relationship between these variables in future research. Second, although the mediating effect was statistically significant, the proportion of mediating effects was relatively small. This may be due to the complexity of the relationships between the variables involved, or the influence of untested external factors. A larger sample size or more nuanced models should be further considered in future studies, which could help to understand better the factors contributing to this limited mediating effect. Third, this study only considered the mediating mechanism of FoMO to affect online social anxiety and did not consider the moderating mechanism in this relationship. Follow-up studies should investigate moderating factors in the relationships between these variables, such as a sense of meaning in life and self-esteem, to provide more comprehensive psychological intervention guidance for university students’ online mental health needs.

## 6. Conclusions

FoMO has a significant positive predictive effect on online social anxiety. Moreover, it not only directly predicts online social anxiety but also indirectly affects online social anxiety through irrational procrastination and media multitasking, which partially mediates the relationship between FoMO and online social anxiety. The model provides an in-depth understanding of how FoMO leads to online social anxiety in university students. It is intended to contribute to research on FoMO, online social anxiety, and other online mental health issues in young adults.

## Figures and Tables

**Table 1:** Descriptive statistics and correlations between variables (N = 451).

Variable	M	SD	1	2	3	4
1. Fear of missing out	2.84	0.75	1			
2. Irrational procrastination	2.97	0.53	0.49 **	1		
3. Media multitasking	0.39	0.17	0.44 **	0.23 **	1	
4. Online social anxiety	2.93	0.76	0.67 **	0.53 **	0.39 **	1

Note: M is the mean value; SD is the standard deviation. ** *p* < 0.01.

**Table 2:** Regression analysis of variable relationships in models (N = 451).

Regression Equation		Fitting Index			Significance	
Result Variable	Predictor Variable	R	R^2^	F	*β*	t
Online social anxiety	Gender	0.68	0.46	126.44 ***	0.09	1.65
	Grade				0.01	0.36
	Fear of missing out				0.69	19.33 ***
Irrational procrastination	Gender	0.50	0.25	48.89 ***	0.09	1.99 *
	Grade				0.001	0.02
	Fear of missing out				0.35	11.98 ***
Media multitasking	Gender	0.46	0.21	40.31 ***	−0.04	−2.98 **
	Grade				0.003	0.43
	Fear of missing out				0.10	10.26 ***
Online social anxiety	Gender	0.72	0.52	97.11 ***	0.08	1.53
	Grade				0.01	−0.31
	Fear of missing out				0.51	12.14 ***
	Irrational procrastination				0.38	6.86 ***
	Media multitasking				0.54	3.17 **

Note: gender: 1 = male; 2 = female. grade: 1 = freshmen; 2 = sophomores; 3 = juniors; 4 = seniors. * *p* < 0.05, ** *p* < 0.01, and *** *p* < 0.001.

**Table 3:** Mediating paths between FoMO and online social anxiety.

Effect	Pathway Relationships	Efficiency Value	Efficiency Value %	95% CI	
				Boot LLCI	Boot ULCI
Direct effect	Fear of missing out → online social anxiety	0.51	74%	0.43	0.59
Indirect effect	Fear of missing out → irrational procrastination → online social anxiety	0.13	19%	0.09	0.17
	Fear of missing out → media multitasking → online social anxiety	0.05	8%	0.02	0.09
Total indirect effect		0.18	26%	0.13	0.24
Total effect		0.69	100	0.62	0.76

Note: LLCI = the lower-limit confidence interval; ULCI = the upper-limit confidence interval.

## Data Availability

The data presented in this study are available on request from the corresponding author.
